# Bridging the Gap: Promoting Interventional Pain Medicine as a Future for Family Physicians

**DOI:** 10.7759/cureus.74841

**Published:** 2024-11-30

**Authors:** Jessan Jishu, Saad Hanan, Farhan Shahid, Alexandra LaForteza, Sanjay Shrestha

**Affiliations:** 1 Family Medicine, Tulane University School of Medicine, New Orleans, USA; 2 Family Medicine, Louisiana State University Health Sciences Center, Alexandria, USA; 3 Surgery, Tulane University School of Medicine, New Orleans, USA

**Keywords:** family med, fellowship training, interventional pain medicine, medical residency, opioids use, primary care medicine, procedural skills training

## Abstract

The use of interventional pain medicine (IPM) has been growing over the past few years, offering relief to patients suffering from acute and chronic pain who have failed conservative therapies. Pain is one of the top complaints that family physicians encounter, yet there is no official pain medicine (PM) fellowship or recognized training program that favors family medicine graduates. Although family medicine residency programs allot a certain amount of time to teaching trainees in PM, this is considerably insufficient and does not dedicate ample time for procedural treatments. This review explains the increasing demand for IPM and provides several arguments for family physicians to provide all aspects of PM, including interventional treatments. It is recommended that pain fellowships recognize family physicians as being ideal candidates for their programs and identify their potential and competencies to perform interventional procedures.

## Introduction and background

Pain medicine (PM) is an evolving subspecialty, with physicians unveiling new methods to mediate pain and promote relief. Although pharmacological management and psychotherapy have significantly reduced pain and restored function, the number of interventional procedures being performed has increased in the past two decades to address pain that has failed conservative measures. Such an increase is quite notable among Medicare beneficiaries, where overall interventional pain services increased by 236% with an annual average of 11% up to 2013 and have been expected to increase further since then [1].

Interventional pain medicine (IPM) has become an integral component of several PM fellowships that welcome physicians from various fields, notably anesthesiology, physiatry, and neurology [2]. Family medicine is also a specialty well-suited to address the multiple dimensions behind chronic pain. Although the American Board of Medical Specialties (ABMS) has made family medicine graduates eligible to apply for PM fellowships, the preponderance of other medical specialties entering these fellowships suggests that there is no training program that openly targets family medicine trainees. In fact, results from the United States National Resident Matching Program (NRMP) showed that only 2.2% of all PM fellows graduated from a family medicine residency in 2023, slightly up from 1.4% the year prior [3]. Family medicine physicians often find chronic pain management difficult due to inadequate training or lack of awareness of pain specialists in the area, just to name a few [4]. With chronic pain being one of the very top reasons that bring patients to visit family physicians, it is worth considering that appropriate training in PM and IPM through fellowship is offered to family medicine trainees. This review describes the growing importance of IPM and sheds light on the partiality of PM fellowships against family medicine, arguing the need for more family physicians to train in IPM.

## Review

Methods

This narrative review involved initial searches of PubMed (NLM), Scopus (Elsevier), and Web of Science Core Collection for articles pertaining to PM, IPM, and the extent to which family physicians learn about and manage pain. Referenced articles within articles found in these initial searches were also reviewed for compatibility and relevance. Three authors (JAJ, SH, FS) independently conducted thorough searches through these databases with no date restrictions and compiled them for further investigation. To maximize the sensitivity of results, no major preset database filters were used. Articles that were not peer-reviewed, not written in the English language, or did not contribute meaningful insight to our research question were excluded. Discrepancies of decisions regarding articles to investigate and use in this review were resolved through extensive discussion.

Overview of IPM

Indications

Interventional procedures provide an approach to relieve pain for chronic pain conditions in situations where lifestyle and medical management have failed. Recent evidence-based reports have concluded that pharmacological treatments for pain are limited, as evidenced by no more than 40-60% of patients experiencing even partial relief of their pain [5].

Furthermore, increasing trends in opioid misuse or non-prescribed opioid usage have influenced a greater interest in procedures to avoid the potential for any substance abuse [1]. The decision as to whether an interventional procedure should be performed (and the specific type of procedure) is made upon various prerequisites and generally after the completion of more conservative treatments [2,4]. However, its limited success in relieving pain and the opioid misuse that results from long-term opioid use has started to influence physicians to incorporate IPM earlier [5].

Patient Perception

IPM has become more popular among healthcare providers and patients over the past few years. Recent studies have revealed high satisfaction scores by patients with various chronic medical conditions experiencing pain undergoing IPM for immediate pain relief [6,7]. Hambraeus et al. found that IPM can also enhance the bond between a patient and their provider, as performing specific procedures cannot be done under image guidance alone but rather requires the cooperation of the patient to evaluate pain throughout the intervention, thus promoting patient empowerment and control over their care [8]. Indeed, IPM serves as a promising treatment option for addressing pain, strengthening the provider-patient relationship, and promoting continuity of care.

Increasing Demand for IPM

Chronic pain has been deemed both a prominent public health and economic problem in the United States. In recent years, national health expenditures have been estimated to be $560-630 billion each year, substantially higher than those for cancer, diabetes, and heart disease combined [9,10]. Although conservative treatments have their benefits, relying exclusively on them to relieve pain is insufficient due to a combination of factors, such as increasing trends of medication non-compliance, drug interactions, regular follow-up appointments and dosing readjustments, and emotional distress and impairment affecting outcomes of therapies [11-13].

Of particular note is opioid therapy, which has been extensively used for chronic pain in the United States. Despite its benefits in managing acute pain, it dramatically increases the risk of exacerbating opioid misuse, which has become a national public health concern. Indeed, overdose-related deaths in the United States reached a peak in 2019, only to have been accelerated by the coronavirus disease 2019 pandemic (COVID-19) and increased by more than 35% since then [14]. Unfortunately, there is limited evidence of the effectiveness of long-term opioid therapy for chronic pain in the literature. Nury et al. performed a comprehensive systematic review and found that opioid therapy use between 12 months and 18 months may not be superior to non-opioid therapy for treating pain or disability, although this conclusion was based on cumulative evidence from randomized control trials and nonrandomized studies of interventions in multiple countries [15]. Manhapra et al. use the notion of negative reinforcement in addiction to suggest that the repeated use of a potentially addictive drug such as an opioid can establish an opposing effect to pain relief and can actually reduce the net relief after administration, motivating patients’ need to enhance their opioid usage with the hope to counteract this limited effectiveness [16]. This mechanism agrees with the notion that patients can become tolerant of their opioid therapy and can increase their doses over time, which carries a greater risk of adverse events [17]. Nevertheless, comparing the effectiveness and pain relief outcomes between patients using long-term opioid and non-opioid therapy warrants further study.

With chronic pain becoming refractory to such conservative measures, IPM is becoming recognized as an effective and popular next step. Recent studies have shown that patients who pursued IPM have achieved meaningful improvements in pain and function, as well as reduced opioid use with very few side effects [18]. This increasing demand has also pushed for procedures to be performed in outpatient settings, citing improvements in procedure accuracy, waiting times, and health costs compared to hospital-based settings [19]. IPM has also earned particular interest from clinicians, influencing them to argue for increased research funding for new interventional therapies [20]. Indeed, its popularity has made it an essential component of PM fellowships where physicians can train in performing procedures.

Training and certification

PM is a multidisciplinary field where multiple residency program graduates can enter for fellowship training. However, innovations in pain treatment modalities have allowed graduates from other residency programs, such as psychiatry or family medicine, to enter. Despite family medicine trainees’ eligibility to apply for PM fellowships, data regarding program directors’ preferences for applicant selection in 2024 revealed that family medicine trainees are preferred the least compared to anesthesiology, emergency medicine, neurology, physiatry, and psychiatry [21].

To date, there are 114 PM fellowship programs accredited by the Accreditation Council for Graduate Medical Education (ACGME), representing an increase of 10.6% in the last five years [3]. Currently, IPM predominantly serves as a major component of pain fellowships rather than existing as its own fellowship. The diversity in clinical training and residency-based backgrounds of fellows makes the beginning of PM fellowships composed of various instructions and experiences. Nevertheless, the Joint Commission (TJC), a United States-based organization responsible for medical service accreditation and credentialing worldwide, has provided clear standards for physicians to be credentialed in PM. Although it has not offered similar expectations for IPM, there exist several academic societies that have recommended certain milestones to ensure competency in performing procedures. Nevertheless, the ACGME has required PM fellows to be competent in performing certain interventional techniques as well as understanding their indications and risks (Table 1) [22].

**Table 1:** Summary of ACGME-required interventional techniques for PM fellows ACGME: Accreditation Council for Graduate Medical Education; PM: pain medicine

Interventional technique
Epidural injections (e.g., interlaminar, transforaminal, caudal)
Trigger point injections
Facet and medial branch blocks
Neuro-ablation
Joint and bursa injections
Sympathetic nerve blocks
Peripheral nerve blocks

The case for family physicians to pursue PM and IPM

Family physicians are designated to organize and lead all aspects of patients’ health, making them ideal personnel to be the central access point in managing their pain [23]. Interestingly, studies reveal that they may even be more aware of current treatments, indications, and best practices for managing pain than other specialists, such as orthopedic surgeons [24]. Furthermore, there is a strong sense of trust between family physicians and their patients, which can be attributed to the continuity of care that they provide. Indeed, patients’ trust in their providers has been shown to be associated with lower pain intensity ratings and improved procedural outcomes [25,26]. Although family physicians do not have the training to perform open or extensive procedures, performing interventional procedures may certainly be within their scope of practice. Family physicians receive high trust and comfort from their patients, suggesting that they have the potential to perform these procedures with favorable outcomes. As such, recent letters have advocated for family practitioners to further train in managing pain [27].

Primary care physicians play essential roles in selecting which patients may be candidates for IPM and referring them to interventional pain specialists, highlighting the importance of collaboration between the two medical specialties regarding pain management [28]. However, having the primary care physician assume sole responsibility for pain management could prevent the need for any referrals in the first place. This is especially important since most primary care and family physicians working in urban and rural settings find it difficult to refer patients due to a shortage of services, long waitlists, or unawareness of PM and IPM specialists in their communities [29]. Intuitively, PM is most effective when pain is addressed through a multidisciplinary approach encompassing other modalities such as psychology and mental health, exercise and physical therapy, pharmaceuticals, and interventional procedures [30]. Appreciating this holistic view, it is also sensible to argue that family medicine physicians, widely known as primary care physicians who care for all aspects of their patients, can also assume all aspects of care for their patient’s pain.

Nevertheless, the majority of family physicians disclose poor professional training for treating chronic pain [31]. Family medicine residency programs report an average of 33 hours of training to address chronic pain, with only 4.2 hours geared toward non-pharmacologic treatment [32]. Studies from the past three years demonstrate that increasing the amount of training allocated toward PM through specialized modules or training improves residents’ competency and confidence in managing pain (Table 2). Indeed, such interventions demonstrate that family physicians can adequately treat pain when the appropriate training is provided. With family physicians being more likely to perform procedures that they have learned, it is essential for training programs to offer more training in interventional procedures as well. Various training programs emphasizing general procedures resulted in excellent feedback from their trainees, who appreciated and recognized the hands-on experiences as catalysts for their competency and confidence [33-35]. Furthermore, prior studies have revealed that there is no evident increase in complications or decrease in the quality of family physicians performing non-complex procedures when compared to specialists [36]. Given that residency is designed to provide trainees with a rich breadth of various specialties and exposure to conditions, PM fellowships should be offered to family medicine graduates. Despite PM’s reputation for being a competitive specialty, the percentage of filled PM fellowship programs has continuously decreased over the past five years [3]. Indeed, welcoming family physicians to claim these unfilled positions can allow them to build upon their foundational skills by focusing on the pharmacological and interventional methods of treating pain.

**Table 2:** Summary of articles describing specialized pain medicine training for family medicine residents and their outcomes

Year	Authors	Journal	Findings
2023	Traxler et al. [37]	Family medicine	Introducing a specialized didactic curriculum improves family medicine providers’ comfort in managing chronic pain and compliance with guidelines from the Center for Disease Control (CDC) regarding drug prescriptions.
2023	Ofei-Dodoo et al. [38]	Family medicine	Participating in a three-month intervention comprised of lectures, clinical examination activities, and briefing with faculty improves first-year residents’ comfort and knowledge of appropriately managing chronic pain with opioids.
2022	Awadakkah et al. [39]	Family medicine	Organizing a committee to design and implement a longitudinal curriculum focusing on chronic pain management enhances resident education in pain management, decreasing the number of opioids prescribed while improving post-treatment pain.
2022	Khera et al. [33]	PRiMER	Forming a procedure clinic rotation increases the number of overall procedures, including joint and soft tissue injections for pain, improving residents’ competency and confidence.
2021	Chiasson et al. [40]	Family medicine	Introducing an online 11-hour integrative pain management course improves residents’ knowledge of pain management, attitude towards patients suffering from pain, and confidence in prescribing non-drug therapies.

Normalizing PM as a potential fellowship and career opportunity for family medicine graduates can attract more physicians to pursue residency in family medicine and practice primary care, a well-known problem in the United States [41]. In fact, the potential of training in PM through fellowship can address many barriers that discourage medical students from pursuing a family medicine residency. One barrier is the notion of family medicine having a poor knowledge base and thus providing little intellectual challenge, incompatible with the belief that there is now a high demand for subspecializing in a field [42-44]. Training in general and interventional pain is notable for precipitating several academic challenges, such as spinal anatomy and mechanics [3,45]. Furthermore, recent literature concluded that family physicians may find improved overall job satisfaction by increasing the number of procedures they perform, suggesting that IPM may provide an outlet for increased contentment [46].

Challenges

Difficulties exist in expanding PM fellowships to family physicians. Obstacles to IPM education, for example, may be attributed to a paucity of faculty to teach interventional procedures or opposition from orthopedic and neurosurgical specialists [3]. Emphasizing the multidisciplinary nature of PM, this issue can possibly be mediated by recruiting faculty from multiple specialties rather than from one. Regarding surgeons’ opposition to the notion of non-surgeon physicians performing interventional procedures, it is recommended that surgeons and non-surgeon providers improve their interprofessional relations through collaboration and research to prioritize the needs of patients rather than professional pride [47]. Interestingly, it has been found that incorporating procedural and intraoperative training into family medicine residencies has equipped residents with the hands-on skills that are in high demand in underserved regions without creating competition between them and surgical trainees [48]. Such training has allowed family physicians to provide clinical and procedural care in areas that may lack access to surgical care, implying that enabling family physicians to perform IPM in these areas may treat patients that will not impinge on the demand for surgeons.

There may also be concern that expanding PM fellowships can induce family physicians to focus on managing pain rather than practicing the entire breadth of primary care. Indeed, fellowships tend to influence physicians to narrow their scope of practice into a single and specialized field. The purpose of a fellowship in PM will certainly allow family physicians to secure more procedural training and experience, but such interventional procedures will complement their ordinary day-to-day practice. In theory, the notion of PM being a multidisciplinary field makes it difficult for family physicians to stray away from their main duties in providing primary care.

Another challenge is the dearth of procedural and outcome-related data regarding family physicians performing IPM. It is recommended that there be further studies to investigate such variables as the time length of the procedure, physician-reported confidence levels, patient pain level and satisfaction post-procedure, and incidence of complications, all of them especially compared to pain physicians trained in other specialties such as anesthesiology or physiatry.

## Conclusions

Family physicians encounter pain on a regular basis. Intuitively, it is essential that they can confidently and competently manage pain in all modalities. Historically, PM fellowships have skewed away from family physicians, preventing them from pursuing further training in the non-interventional and interventional means of treating pain. With PM evolving as a field and gradually involving IPM in treatment, so too must the group of specialists practicing it. Family physicians are holistic providers of care and should be accountable for addressing all aspects of their patients' pain, only referring them to other specialists when their non-interventional and interventional treatments have failed.

PM fellowships should reduce their bias toward other specialties and welcome family medicine as an ideal field to further specialize in pain. This expansion can stimulate PM into an important field that combines the important tenets of multiple specialties into one, which can motivate further training, research, and practice. Furthermore, doing so can prepare more physicians to treat one of the most prevalent public health issues around the world.
